# Evaluation of Hepatic Detoxification Effects of *Enteromorpha prolifera* Polysaccharides against Aflatoxin B_1_ in Broiler Chickens

**DOI:** 10.3390/antiox11091757

**Published:** 2022-09-06

**Authors:** Wen-Chao Liu, Yu-Ying Yang, Karthika Pushparaj, Balamuralikrishnan Balasubramanian

**Affiliations:** 1 Department of Animal Science, College of Coastal Agricultural Sciences, Guangdong Ocean University, Zhanjiang 524088, China; 2 Department of Zoology, School of Biosciences, Avinashilingam Institute for Home Science and Higher Education for Women, Coimbatore 641 043, Tamil Nadu, India; 3 Department of Food Science and Biotechnology, College of Life Science, Sejong University, Seoul 05006, Korea

**Keywords:** aflatoxin B_1_, hepatotoxicity, detoxification, seaweed polysaccharides, *Enteromorpha prolifera*, natural antioxidants

## Abstract

Aflatoxin B_1_ (AFB_1_) is a major risk factor in animal feed. Seaweed (*Enteromorpha prolifera*)-derived polysaccharides (SDP) are natural antioxidants with multiple biological functions, which may have an in vivo detoxification effect on AFB_1_. The current study aimed to evaluate whether SDP could mitigate AFB_1_-induced hepatotoxicity in broilers. A total of 216 chickens (male, 5 weeks old) were randomly allocated to three groups with differing feeding patterns, lasting 4 weeks: (1) control group (CON, fed a basal diet); (2) AFB_1_ group (fed a basal diet mixed with 0.1 mg/kg AFB_1_); and (3) AFB_1_ + SDP group (AFB_1_ group + 0.25% SDP). The results showed that dietary SDP improved the liver function-related biochemical indicators in serum, and reversed the increase in relative liver weight, hepatic apoptosis and histological damage of broilers exposed to AFB_1_. SDP treatment also reduced the activity and mRNA expression of phase I detoxification enzymes, while increasing the activity and mRNA expression of phase II detoxification enzymes in the livers of AFB_1_-exposed broilers, which was involved in the activation of p38 mitogen-activated protein kinase (p38MAPK)/nuclear factor erythroid 2-related factor 2 (Nrf2) signaling. In conclusion, dietary SDP alleviated AFB_1_-induced liver injury of broilers through inhibiting phase I detoxification enzymes and upregulating p38MAPK/Nrf2-mediated phase II detoxification enzymes pathway.

## 1. Introduction

At present, chicken is a major meat consumer product worldwide, and the food safety of chicken is critical to public health [1]. Feed toxins are easily transmitted to humans through the food chain during chicken production [2]. The contamination of mycotoxins in feed is a significant risk factor for broiler production and food safety [3]. Grains and other feed materials breed mold and produce mycotoxins during production procedures, transportation and storage, and more than 300 kinds of mycotoxins have been found [4]. Among these mycotoxins, aflatoxins are highly toxic secondary metabolites produced by *Aspergillus flavus* and *A. parasiticus* [5]. The diverse derivatives of aflatoxins have been identified, including aflatoxin B_1_, B_2_, G_1_, G_2_, M_1_ and M_2,_ etc., and the aflatoxin B_1_ (AFB_1_) is classified as a class I carcinogen because it has the strongest toxicity and the widest distribution [6]. The AFB_1_ accumulation in broiler muscle was found to be mediated by dietary intake, which leads to chicken meat contamination and endangers human health [7]. 

The liver is the AFB_1_ detoxification organ, and it is also the main target organ for the toxic effects of AFB_1_ in chickens [8]. Broiler chickens are highly susceptible to AFB_1_. According to Chinese feed hygiene standards, the maximum limit of AFB_1_ is set at 10 μg/kg for juvenile chickens and 20 μg/kg for adult chickens; exceeding the maximum limit will damage the health status of broilers [9]. However, China is a large-scale chicken producing and consuming country, and among 175 feed ingredients collected from different regions, the detection rate of AFB_1_ was found to reach 94.9%, and the AFB_1_ content of some samples reached 50–100 μg/kg [10]. Numerous direct pieces of evidence demonstrated that broiler chickens exposed to greater than 50 μg/kg AFB_1_ exhibited reduced performance, morphological damage and physiological dysfunction of the liver [11,12,13,14,15]. 

The bioactivation of AFB_1_ are mainly dependent on the phase I detoxification enzyme system (cytochrome P450, CYP450) [6]. AFB_1_ is metabolized to AFB_1_-8,9-epoxide (AFBO)-DNA by phase I detoxification enzymes, which triggers hepatotoxicity and oxidative damage to liver [16]. On the contrary, phase II detoxification enzymes (e.g., glutathione S-transferase, GST; glutathione peroxidase, GSH-Px) promote the metabolism and excretion of AFBO-DNA and alleviate oxidative stress, thereby attenuating AFB_1_-induced hepatotoxicity [17].

There are a rising number of studies indicating that natural antioxidants can prevent AFB_1_ hepatotoxicity by targeting phase I and/or phase II detoxification enzymes in broilers [18]. For instance, curcumin has been reported to alleviate AFB_1_-induced liver injury of chickens; this beneficial effect was achieved via inhibition of CYP450 isozymes, and activation of nuclear factor erythroid 2-related factor-2 (Nrf2) involved GSTs pathway [19,20,21,22,23]. Lycopene and proanthocyanidins also exerted protective effects against AFB_1_ hepatotoxicity and showed similar mechanisms of action to curcumin [24,25,26,27]. Therefore, the phytoproducts’ combatting of AFB_1_ toxicity may be mainly owed to their antioxidant function [18]. 

*Enteromorpha prolifera* is a green seaweed with abundant sources, which is widely distributed in the East Asian Ocean Belt [28]. *E. prolifera* has long been used as a traditional medicinal plant in China. The polysaccharide is the main bioactive component, and has health benefits for humans [29]. It has been proven that seaweed-derived polysaccharides (SDP) from *E. prolifera* exhibit various biological activities, such as antioxidant, hepatoprotective and anti-inflammatory activities [29,30,31,32,33]. Previous reports suggested that SDP could improve the antioxidant capacity and upregulate phase II detoxification enzyme gene expression through modulating the Nrf2 signaling pathway in broilers [34,35]. A more recent study demonstrated that SDP mitigated immune organ injury caused by AFB_1_ exposure by regulating the p38MAPK/Nrf2-mediated phase II detoxification enzyme signaling pathway [36]. These findings indicate that SDP has the potential to act as a decontamination agent of AFB_1_ in vivo. However, to date, no report has defined the protective effect of SDP against hepatotoxicity in broiler chickens fed an AFB_1_-contaminated diet. Therefore, this study was conducted to investigate the effects of SDP in attenuating AFB_1_-induced liver injury of chickens, and to uncover the underlying mechanism.

## 2. Materials and Methods

### 2.1. Animals and Experimental Design

A total of two hundred and sixteen yellow-feathered broilers (five weeks old, male) were obtained from the local producers and used in this study. The chickens were randomly split into three treatment groups: (1) control group (CON, provided basal diet); (2) AFB_1_ group (basal diet mixed with 0.1 mg/kg AFB_1_); (3) AFB_1_ + SDP group (AFB_1_ group + 0.25% SDP). The duration of this animal study was four weeks. The AFB_1_ was obtained from a commercial reagents company (Sigma-Aldrich Co., Ltd., St. Louis, MO, USA). Each treatment group was subjected to six replicate cages (12 broilers/replicate cage). The SDP products were extracted from the seaweed *E. prolifera*, the details of SDP such as purity, chemical composition, structure and molecular weight, were analyzed in our previous report [31] and the chemical and monosaccharide composition of SDP was described in Table S1. The environmental conditions and basal diet composition (including the AFB_1_ content in basal diet) were also referred to in our earlier study [36]; the formulation and nutrient levels of the basal diet were presented in Table S2.

### 2.2. Growth Performance and Sampling

During the feeding trail, feed intake was monitored daily on a per-cage basis for the detection of average daily feed intake (ADFI). At the end of the animal experiment, the body weights of broilers were measured per cage, and then the average daily gain (ADG) and the ratio of feed/gain (F/G) were calculated.

One bird was selected randomly from each replicate cage at the end of the animal study for sampling (n = 6 per group). These birds were weighed individually, and the blood samples were collected into vacuum tubes without anticoagulants via subwing veins. The relative liver weight was determined as previously reported [19]. The coagulated bloods were centrifuged to collect serum samples for a biochemical parameters assay (3000 r/min, 5 min). Then, the chickens were slaughtered by neck bleeding, and the livers were separated, rinsed with saline and weighed. Subsequently, the liver tissues were fixed with 4% paraformaldehyde for histological, apoptosis and immunohistochemical analysis. Other liver samples were stored at –80 °C until further analysis of enzyme activity, mRNA and protein expression.

### 2.3. Serum Biochemical Analysis

The activity of alanine transaminase (ALT) and aspartate transaminase (AST), and the total protein levels in serum, was determined using the Chemray 800 Automatic Biochemical Analyzer (Redu Life Technology Co., Ltd., Shenzhen, China).

### 2.4. Hepatic Histopathology and Apoptosis Analysis

The preparation and observation of histological sections and histopathological analysis were based on our previous report [36]. The TdT-mediated dUTP Nick-End Labeling (TUNEL) method was used to detect hepatic apoptosis using the paraffinized sections; the details of the TUNEL method are as we previously reported [33]. Green indicates apoptosis-positive cells in the figures.

### 2.5. Determination of Enzymatic Activity and AFBO-DNA Content of Liver

The activity of total-superoxide dismutase (T-SOD), GSH-Px, catalase (CAT) and GST and the content of malondialdehyde (MDA) in the liver were analyzed using commercial kits from the Jiancheng Institute (Nanjing, China) following the manufacturer’s instructions. The activity of heme oxygenase-1 (HO-1) and CYP450, and the content of AFBO-DNA, were analyzed using the commercial kits (Jiangsu Enzyme Immunology Co., Ltd., Suzhou, China), according to the manufacturer’s instructions.

### 2.6. Gene Expression Analysis of Liver

The reagents and protocols of RNA extraction, cDNA transcription and quantitative real-time polymerase chain reaction (qPCR) reaction were described in our previous report [33]. The internal control gene was the *β-actin.* In addition, the information of used primers was based on the former studies [19,33]. The method of 2^−ΔΔCt^ was used for data processing [37], and the relative mRNA expression compared with the CON group was used to express the gene expression results.

### 2.7. Immunohistochemical Analysis and Western Blotting

Immunohistochemistry was performed to determine the Nrf2 protein expression in the liver samples, in accordance with the earlier report [24]. The protein expression of p38MAPK, HO-1, Nrf2, total-Nrf2 and nuclear-Nrf2 was determined using Western blotting; detailed methods and information on antibodies have been described previously [33,36]. The immunohistochemical results were expressed as areal density, and the Western blotting results were expressed as relative protein expression (target protein/β-actin).

### 2.8. Statistical Analysis

The general linear model procedure in SAS 9.4 (SAS, 2013. SAS Institute Inc., Cary, NC, USA) was used to process and analysis the data in this study. The analysis of variance was used for significance testing, and then the significant differences among the three treatment groups were determined using Tukey’s test. *p* < 0.05 was considered statistically significant.

## 3. Results

### 3.1. Growth Performance, Serum Biochemical Indicators and Relative Liver Weight

As presented in Table 1, AFB_1_ exposure and SDP treatment had no significant impacts on ADG, ADFI and F/G of chickens (*p* > 0.05). The results of serum biochemical indicators and relative liver weight are shown in Table 2. Compared with the CON group, chickens fed an AFB_1_-contaminated diet showed increased serum ALT and AST activity (*p* < 0.05), and an elevated relative liver weight (*p* < 0.05). Dietary SDP could reduce the serum ALT and AST activity (*p* < 0.05) and decrease the relative liver weight (*p* < 0.05) in broilers after being subjected to AFB_1_ exposure.

### 3.2. Histopathology and Apoptosis of Liver

**Figure 1:**
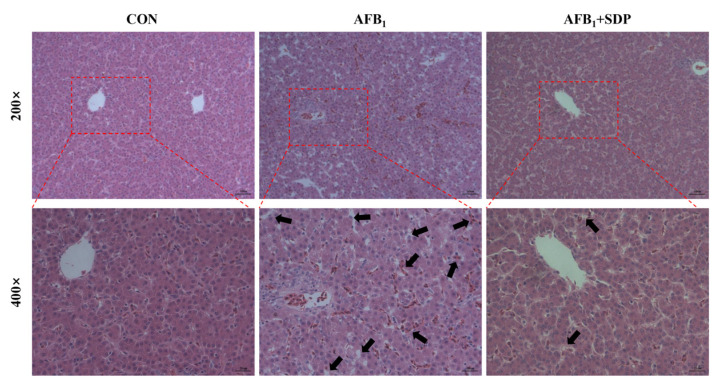
The protective effects of dietary seaweed (*Enteromorpha prolifera*)-derived polysaccharides on histopathological changes in liver in broiler chickens. Scale bars for 200× are 50 μm and 400× are 25 μm. Arrows indicate the vacuolization, inflammatory cell infiltration and hepatocyte necrosis.

**Figure 2:**
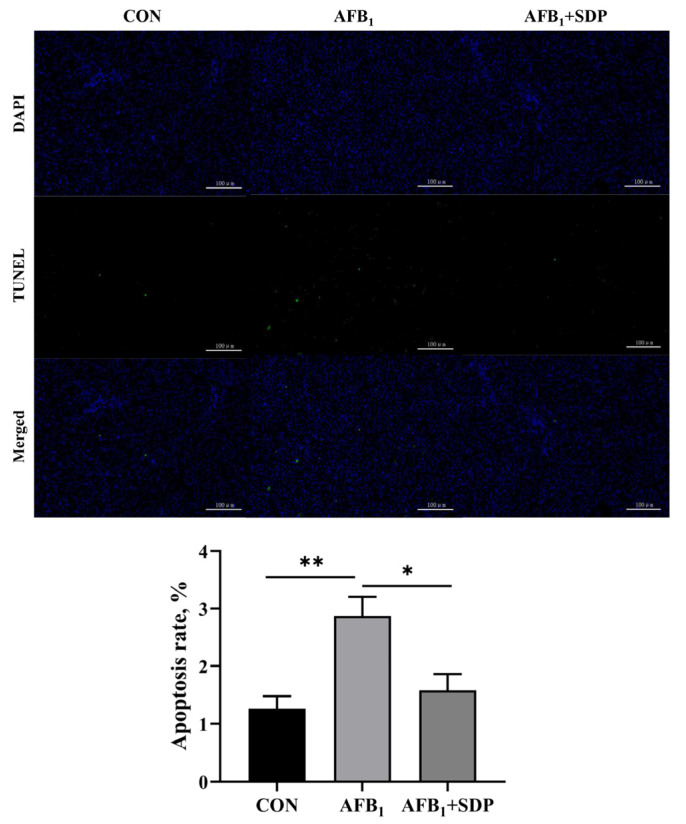
The protective effects of dietary seaweed (*Enteromorpha prolifera*) derived polysaccharides on apoptosis of hepatocytes in broiler chickens. The scale bar is 100 μm. * *p* < 0.05, ** *p* < 0.01, no superscript marks indicated that *p* > 0.10. CON, control group; AFB_1_ group (fed diet with 0.1 mg/kg AFB_1_); AFB_1_ + SDP group (AFB_1_ group + 0.25% SDP).

The hepatic histopathology is illustrated in Figure 1. There were no histopathological alterations of liver in the CON group. Vacuolization, inflammatory cell infiltration and hepatocyte necrosis were observed in the AFB_1_ group. Dietary SDP restored the AFB_1_-induced histopathological changes in the liver. As shown in Figure 2, compared with the CON group, the AFB_1_ group had a higher apoptosis rate in hepatocytes (*p* < 0.01), while SDP intervention reduced the apoptosis rate in hepatocytes (*p* < 0.05).

### 3.3. Enzymatic Activity and AFBO-DNA level of Liver

**Figure 3:**
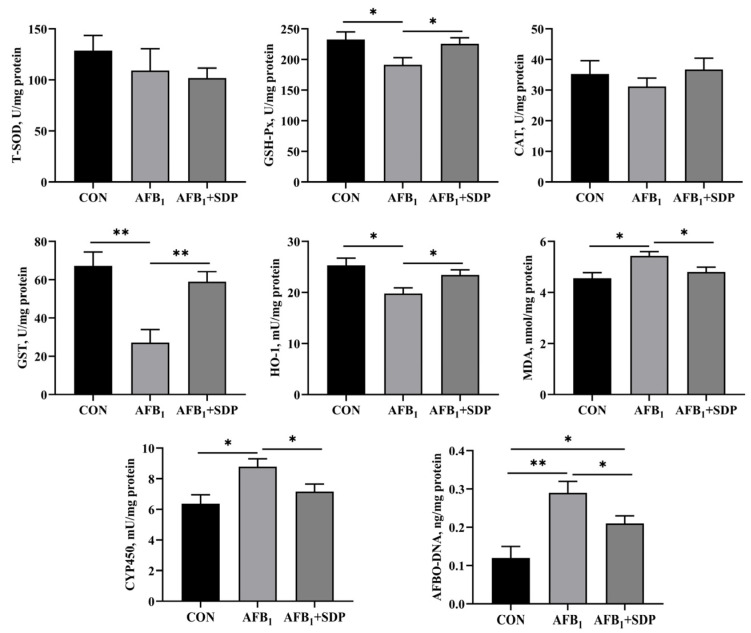
The protective effects of dietary seaweed (*Enteromorpha prolifera*)-derived polysaccharides on enzymatic activity and AFBO-DNA level in liver of broiler chickens. * *p* < 0.05, ** *p* < 0.01, no superscript marks indicated that *p* > 0.10. CON: control group; AFB_1_ group (fed diet with 0.1 mg/kg AFB_1_); AFB_1_ + SDP group (AFB_1_ group + 0.25% SDP).

The results of enzyme activity and AFBO-DNA content in the liver are shown in Figure 3. Chickens exposed to AFB_1_ had lower activity of GSH-Px, GST and HO-1 (*p* < 0.05), while showing higher activity of CYP450 and MDA and AFBO-DNA content in the liver (*p* < 0.05). Dietary SDP intervention promoted the activity of GSH-Px, GST and HO-1 (*p* < 0.05) and reduced the activity of CYP450 and the content of MDA and AFBO-DNA in the liver of broilers after being fed an AFB_1_-contaminated diet (*p* < 0.05).

### 3.4. Hepatic mRNA Expression of Phase I Detoxification Enzyme-Related Genes

**Figure 4:**
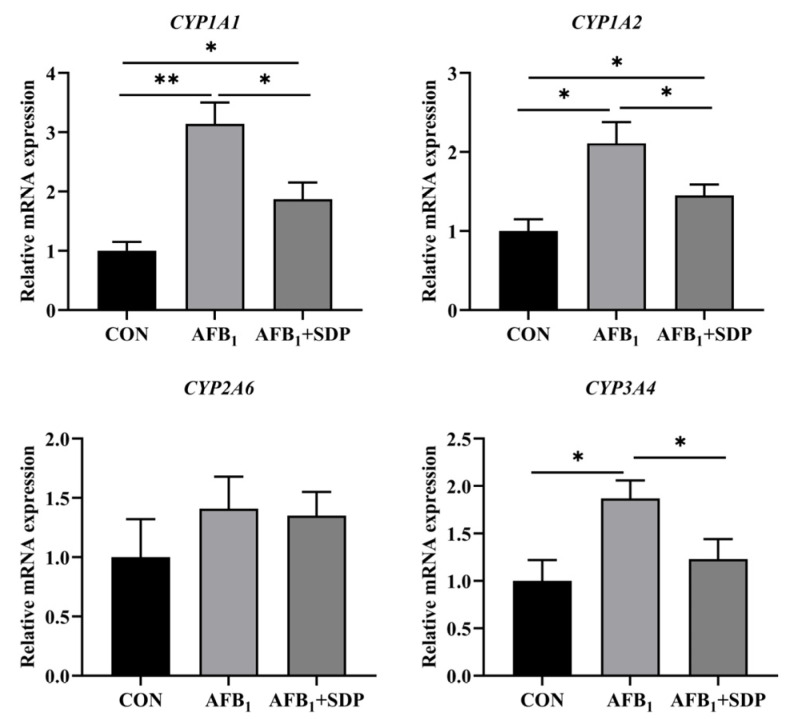
The protective effects of dietary seaweed (*Enteromorpha prolifera*)-derived polysaccharides on hepatic mRNA expression of phase I detoxification enzyme-related genes in broiler chickens. * *p* < 0.05, ** *p* < 0.01, no superscript marks indicated that *p* > 0.10. CON: control group; AFB_1_ group: (fed diet with 0.1 mg/kg AFB_1_); AFB_1_ + SDP group (AFB_1_ group + 0.25% SDP).

As depicted in Figure 4, compared with the CON group, AFB_1_ exposure upregulated the relative mRNA expression of *CYP1A1, CYP1A2* and *CYP3A4* in the liver (*p* < 0.05). Conversely, dietary SDP downregulated the relative mRNA expression of *CYP1A1, CYP1A2* and *CYP3A4* in the liver of broilers exposed to AFB_1_ (*p* < 0.05). When compared to the CON group, the AFB_1_ + SDP group showed a higher relative mRNA expression of *CYP1A1* and *CYP1A2* in the liver (*p* < 0.05).

### 3.5. Hepatic mRNA Expression of Antioxidant and Phase II Detoxification Enzyme Related Genes

**Figure 5:**
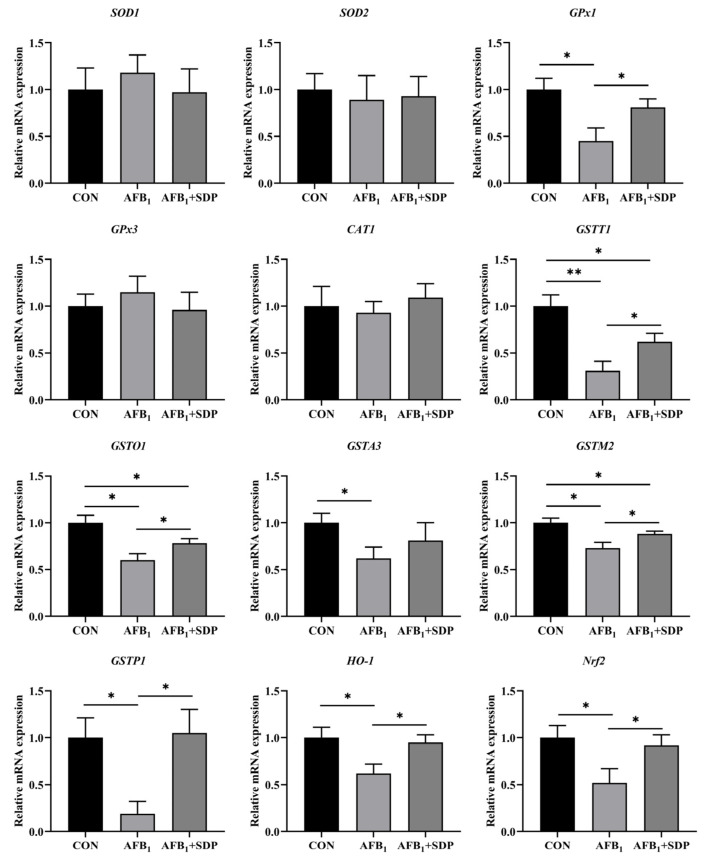
The protective effects of dietary seaweed (*Enteromorpha prolifera*)-derived polysaccharides on hepatic mRNA expression of antioxidant phase II detoxification enzyme-related genes in broiler chickens. * *p* < 0.05, ** *p* < 0.01, no superscript marks indicated that *p* > 0.10. CON: control group; AFB_1_ group: (fed diet with 0.1 mg/kg AFB_1_); AFB_1_ + SDP group (AFB_1_ group + 0.25% SDP).

As shown in Figure 5, compared with the CON group, AFB_1_ exposure downregulated the relative mRNA expression of hepatic *GPx1, GSTT1, GSTO1, GSTA3, GSTM2,* *GSTP1, HO-1* and *Nrf2* (*p* < 0.05). Compared to the AFB_1_ group, the relative mRNA expression of hepatic *GPx1, GSTT1, GSTO1, GSTM2, GSTP1, HO-1* and *Nrf2* was elevated in the AFB_1_ + SDP group (*p* < 0.05). Even then, broilers in the AFB_1_ + SDP group had a lower relative mRNA expression of hepatic *GSTT1, GSTO1* and *GSTM2* than those in the CON group (*p* < 0.05).

### 3.6. Protein Expression of p38MAPK/Nrf2 Signaling Pathway in the Liver

**Figure 6:**
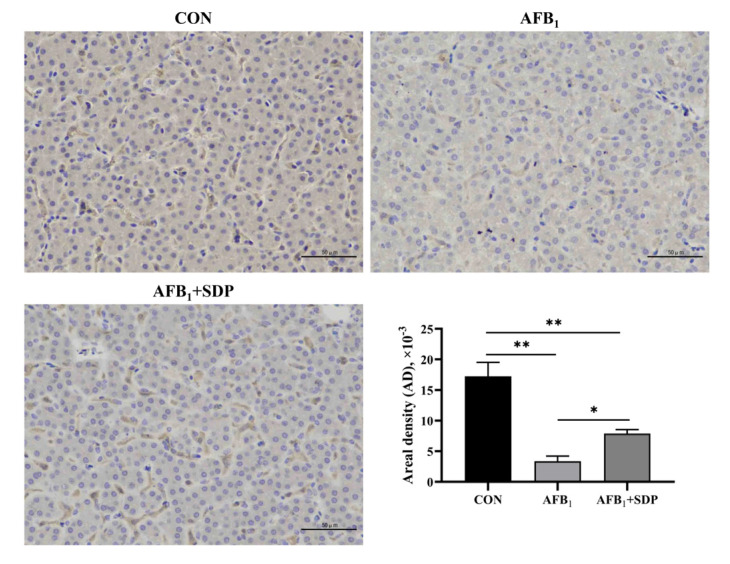
The protective effects of dietary seaweed (*Enteromorpha prolifera*)-derived polysaccharides on hepatic Nrf2 protein expression in broiler chickens based on immunohistochemical analysis. * *p* < 0.05, ** *p* < 0.01, no superscript marks indicated that *p* > 0.10. CON: control group; AFB_1_ group (fed diet with 0.1 mg/kg AFB_1_); AFB_1_ + SDP group (AFB_1_ group + 0.25% SDP).

The results of Nrf2 immunohistochemistry are shown in Figure 6. Compared to the CON group, broilers in the AFB_1_ and AFB_1_ + SDP groups had lower protein expression levels of Nrf2 in the liver (*p* < 0.01). Compared to the AFB_1_ group, dietary SDP supplementation improved the protein expression level of Nrf2 in the liver (*p* < 0.05).

**Figure 7:**
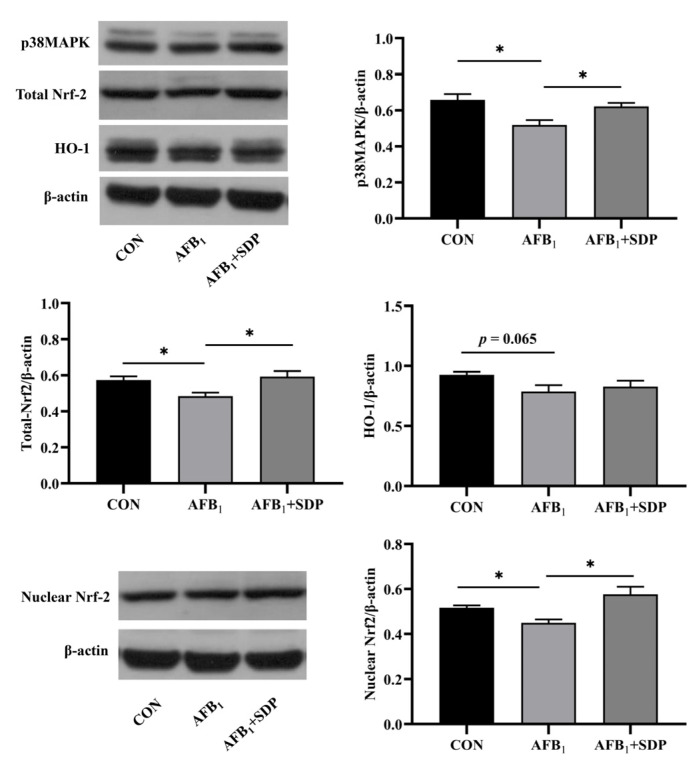
The protective effects of dietary seaweed (*Enteromorpha prolifera*)-derived polysaccharides on hepatic protein expression of p38MAPK, HO-1, total Nrf2, and nuclear Nrf2 in broiler chickens. * *p* < 0.05, no superscript marks indicated that *p* > 0.10. CON: control group; AFB_1_ group (fed diet with 0.1 mg/kg AFB_1_); AFB_1_ + SDP group (AFB_1_ group + 0.25% SDP).

The Western blotting results are presented in Figure 7. AFB_1_ exposure reduced the protein expression level of p38MAPK, total Nrf2 and nuclear Nrf2 in the liver (*p* < 0.05). Inclusion of SDP in the diet could increase the protein expression level of p38MAPK, total Nrf2 and nuclear Nrf2 in the liver (*p* < 0.05).

## 4. Discussion

Prolonged consumption of an AFB_1_-contaminated diet is not only detrimental to broiler health and production, but also causes residue in chicken meat, threatening human health [9]. In production practice, there is typically 20–100 μg/kg AFB_1_ contamination in feed [10]. Accordingly, this study defined 0.1 mg/kg as the toxic dose of AFB_1_ in broilers. The results showed that 0.1 mg/kg AFB_1_ and 0.25% SDP treatment had no significant impacts on the growth performance of broilers, which is similar to a previous study [38]. Existing research reported that AFB_1_ exposure above 0.3 mg/kg significantly reduced the growth performance of broiler chickens [14,39]. Although the production performance was not affected by AFB_1_, it was observed that the serum ALT and AST activity and the relative liver weight were increased in broilers after being subjected to AFB_1_ exposure. The liver is considered to be the main target organ for AFB_1_ attack; relevant studies also confirmed that AFB_1_-exposed chickens had a higher liver index and increased serum ALT and AST activity [19,40,41]. It is well known that ALT and AST are commonly used as clinical biomarkers of liver function; the presence of elevated ALT and AST activity indicates abnormal liver function [42]. The increased liver index might be ascribed to AFB_1,_ resulting in inflammatory enlargement of the liver and/or metabolic disordered lipid accumulation [9]. AFB_1_ also caused histopathological changes and an increase in apoptosis of the liver, which is consistent with the findings of former studies [13,20,40,43], suggesting that AFB_1_ induced liver injury in broilers. 

Interestingly, dietary SDP supplementation improved the impairment of these liver parameters in AFB_1_-exposed broilers. Similarly, Zhang et al. [44] found that dietary natural polysaccharides play a role in hepatoprotection by improving histomorphology and reducing apoptosis of the liver, as well as decreasing the activity of ALT and AST in serum of mice exposed to toxins. Solis-Cruz et al. [41] demonstrated that supplementation of cellulosic polymers mitigated AFB_1_-induced histological disruption and elevation of serum ALT and AST activity in broiler chickens. Feed including other phytoproducts, such as curcumin and lycopene, also showed protective effects on hepatic histopathology and serum ALT and AST, thus preventing the liver damage from AFB_1_ exposure in chickens [19,20,23,24,26]. It is worth mentioning that these effective protection agents are natural antioxidants. Therefore, it is reasonable to speculate that SDP alleviates AFB_1_-induced liver injury, likely due to their antioxidant properties. The specific mechanisms of action are yet to be elucidated.

AFB_1_ bioactivation is depends on the phase I detoxification enzymes (CYP450 isozymes); CYP450 catalyzes the formation of toxic AFBO from AFB_1_ in the liver, which further forms the toxic adduct AFBO-DNA, thereby impairing liver function [6]. Additionally, CYP450 isozymes (namely, CYP1A1, CYP1A2, CYP2A6 and CYP3A4) are responsible for AFB_1_ bioactivation in avian species [9]. Therefore, reducing the activity of CPY450 isozymes can inhibit the bioactivation of AFB_1_ and attenuate the hepatotoxicity of AFB_1_. In this study, broilers in AFB_1_ group had a higher hepatic AFBO-DNA level, CYP450 activity and mRNA expression of *CYP1A1, CYP1A2* and *CYP3A4.* Consistently, extensive research has confirmed that AFB_1_ exposure increases the content of AFBO-DNA and the activity of CYP450 isozymes in chickens’ livers, indicating the bioactivation of AFB_1_ [13,14,15]. It is worth mentioning that our study observed that SDP supplementation decreased the AFBO-DNA content and CYP450 activity and downregulated *CYP1A1, CYP1A2* and *CYP3A4* mRNA expression in the liver of chickens fed an AFB_1_-contaminated diet. Similar to our studies, Gan et al. [45] reported that the polysaccharides from bush sophora root downregulated *CYP1A5* expression, which, in turn, helped to prevent AFB_1_-induced hepatotoxicity in primary chicken hepatocytes. Zhao et al. [39] demonstrated that dietary polysaccharide-rich medicinal plants decreased the AFBO-DNA content of the livers of AFB_1_-exposed broilers. Additionally, other dietary natural phytochemicals showed an inhibitory effect on CYP450-involved bioactivation of AFB_1_ into AFBO. For instance, Zhang et al. [20] found that curcumin reduced the hepatic activity of CYP1A1, CYP1A2, CYP2A6 and CYP3A4, thus inhibiting the production of toxic adduct AFBO-DNA in broilers exposed to AFB_1_. However, the exact mechanism by which natural antioxidants regulate CYP450-mediated AFB_1_ bioactivation remains unclear. In-depth research is imperative to elucidate the mode of action of dietary SDP with respect to CYP450.

**Figure 8:**
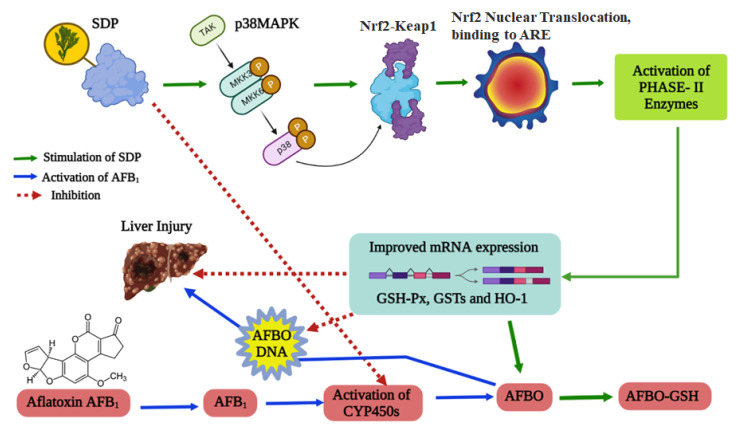
Proposed model of the protective effects of seaweed (*Enteromorpha prolifera*)-derived polysaccharides against aflatoxin B_1_-induced hepatotoxicity in broiler chickens.

AFB_1_-induced broiler liver injury is closely related to oxidative stress and phase II detoxification enzymes, both of which are regulated by the Nrf2 signaling pathway [46]. Nrf2 is a key transcription factor that responds to oxidative stress and regulates phase II detoxification enzymes, and is a sensor of exogenous toxic substances and oxidative stress [47]. Nrf2 is a basic leucine bZip transcription factor; under normal physiological conditions, Nrf2 binds to Kelch-like ECH-associated protein 1 (Keap1) and anchors it in the cytoplasm (Nrf2/Keap1), so that Nrf2 is in a non-free inactive state [46]. Nrf2 can be activated by bioactive compounds and released to undergo nuclear translocation into the nucleus, and it modulates the gene expression of phase II detoxification enzymes (e.g., GSH-Px, GSTs, HO-1), thereby promoting the detoxification process [48]. The Nrf2-mediated phase II detoxification enzyme pathway is also regulated by MAPKs, a class of protein kinases including the subclasses of p38MAPK. Existing studies have confirmed that p38MAPK promoted Nrf2 activation and nuclear translocation and upregulated the expression of downstream phase II detoxification enzymes [49]. The phase II detoxification enzymes can enhance the combination of GSH and AFBO and excrete the AFBO from the body [16]. In the current study, it was found that dietary SDP improved the activity of GSH-Px, GSTs and HO-1 and their corresponding mRNA expression, upregulated the protein expression of p38MAPK and Nrf2 and promoted nuclear translocation of Nrf2 in the liver of broilers after being fed an AFB_1_ diet. Similarly, Guo et al. [36] suggested that dietary SDP alleviated chicken bursal damage triggered by AFB_1,_ which was related to the upregulated mRNA expression of GSTs and the activation of the p38MAPK/Nrf2 signaling pathway. Liu et al. [35] reported that dietary SDP could improve the gut mRNA expression of *GPx1, GSTT1* and *HO-1* by activating the Nrf2 signaling pathway in broilers. Wang et al. [50] demonstrated that dietary natural antioxidants could relieve the uterine injury caused by toxins in chickens and was associated with the activation of the p38MAPK/Nrf2-mediated phase II detoxification enzyme pathway. Meanwhile, other well-known natural antioxidants, such as medicinal plants, curcumin and lycopene, have been reported to alleviate AFB_1_-induced hepatotoxicity by activating the Nrf2-mediated phase II detoxification enzyme pathway [19,22,25,39]. Therefore, it can be concluded that, in addition to suppressing the bioactivation of AFB_1_ by inhibiting CYP450 isozymes, dietary SDP could also activate the phase II detoxification enzyme pathway involved in p38MAPK/Nrf2 signaling, thereby ultimately promoting the hepatic detoxification of AFB_1_ in broilers (Figure 8).

## 5. Conclusions

To summarize, dietary SDP improved the liver-function-related biochemical indicators in serum and reversed the increase in the relative liver weight of broilers exposed to AFB_1_. Histological and apoptosis results suggested that AFB_1_-induced liver injury was partially ameliorated by SDP supplementation. Additionally, SDP treatment enhanced the activity of GSH-Px and GST while reducing the activity of CYP450, MDA and AFBO-DNA levels in the liver of broilers exposed to AFB_1_. Dietary SDP downregulated the hepatic mRNA expression of *CYP1A1, CYP1A2* and *CYP3A4* in broilers after being subjected to AFB_1_ exposure. Moreover, SDP supplementation upregulated the hepatic mRNA expression of *GPx1, GSTT1, GSTO1, GSTP1* and *GSTM2* in AFB_1_-exposed broilers, and this regulation was involved in the activation of p38MAPK/Nrf2 signaling. Therefore, this study revealed, for the first time, that dietary SDP from *E. prolifera* promoted hepatic detoxification of AFB_1_ by inhibiting phase I detoxification enzymes and upregulating the p38MAPK/Nrf2-mediated phase II detoxification enzymes pathway, which provided a reference and a mechanistic basis for the use of SDP as a novel antioxidant and AFB_1_ decontamination agent in broiler chickens.

## Figures and Tables

**Table 1:** The impacts of polysaccharides from seaweed (*Enteromorpha prolifera*) on growth performance of broiler chickens exposed to AFB_1_.

Items	CON	AFB_1_	AFB_1_ + SDP	SEM	*p*-Value			
					ANOVA	CON vs. AFB_1_	AFB_1_ vs. AFB_1_ + SDP	CON vs. AFB_1_ + SDP
ADG, g	21.34	20.77	21.06	0.41	0.629	0.348	0.626	0.640
ADFI, g	75.05	75.42	73.96	1.31	0.732	0.846	0.458	0.579
F/G	3.51	3.63	3.54	0.04	0.152	0.089	0.098	0.928

ADG: average daily gain; ADFI: average daily feed intake; F/G: the ratio of feed/gain; SDP: seaweed-derived polysaccharides; SEM: standard error of the mean; ANOVA: analysis of variance. CON: control group; AFB_1_ group: fed 0.1 mg/kg AFB_1_; AFB_1_ + SDP group: fed AFB_1_ + 0.25% SDP.

**Table 2:** The impacts of polysaccharides from seaweed (*Enteromorpha prolifera*) on serum liver function-related indexes and relative liver weight of broiler chickens exposed to AFB_1_.

Items	CON	AFB_1_	AFB_1_ + SDP	SEM	*p*-Value			
					ANOVA	CON vs. AFB_1_	AFB_1_ vs. AFB_1_ + SDP	CON vs. AFB_1_ + SDP
ALT activity, U/L	6.68	8.05	6.82	0.35	0.038	0.020	0.033	0.782
AST activity, U/L	232.72	272.59	249.48	6.60	0.005	0.002	0.032	0.103
TP content, g/L	40.55	44.28	43.01	1.49	0.243	0.106	0.558	0.269
Relative liver weight, %	2.06	2.35	2.12	0.07	0.049	0.020	0.045	0.683

ALT: alanine transaminase; AST: aspartate transaminase; TP: total protein; SDP: seaweed-derived polysaccharides; SEM: standard error of the mean; ANOVA: analysis of variance. CON: control group; AFB_1_ group: fed 0.1 mg/kg AFB_1_; AFB_1_ + SDP group: AFB_1_ + 0.25% SDP.

## Data Availability

Not applicable.

## Supplementary Materials

The following supporting information can be downloaded at: https://www.mdpi.com/article/10.3390/antiox11091757/s1, Table S1: The chemical and monosaccharide composition of seaweed-derived polysaccharides (SDP) from *Enteromorpha prolifera*; Table S2: The formulation and nutrient levels of the basal diet.

## References

[1] Maharjan P., Martinez D., Weil J., Suesuttajit N., Umberson C., Mullenix G., Hilton K., Beitia A., Coon C. (2021). Physiological growth trend of current meat broilers and dietary protein and energy management approaches for sustainable broiler production. Animals.

[2] Ochieng P.E., Scippo M.-L., Kemboi D.C., Croubels S., Okoth S., Kang’ethe E.K., Doupovec B., Gathumbi J.K., Lindahl J.F., Antonissen G. (2021). Mycotoxins in Poultry Feed and Feed Ingredients from Sub-Saharan Africa and Their Impact on the Production of Broiler and Layer Chickens: A Review. Toxins.

[3] den Hollander D., Croubels S., Lauwers M., Caekebeke N., Ringenier M., De Meyer F., Reisinger N., Van Immerseel F., Dewulf J., Antonissen G. (2021). Applied Research Note: Biomonitoring of mycotoxins in blood serum and feed to assess exposure of broiler chickens. J. Appl. Poult. Res..

[4] Ogbuewu I. (2011). Effects of mycotoxins in animal nutrition: A review. Asian J. Anim. Sci..

[5] Agag B. (2004). Mycotoxins in foods and feeds: 1-aflatoxins. Ass. Univ. Bull. Environ. Res..

[6] Guengerich F.P., Johnson W.W., Shimada T., Ueng Y.-F., Yamazaki H., Langouët S. (1998). Activation and detoxication of aflatoxin B1. Mutat. Res. Fundam. Mol. Mech. Mutagenesis.

[7] Hussain Z., Khan M.Z., Khan A., Javed I., Saleemi M.K., Mahmood S., Asi M.R. (2010). Residues of aflatoxin B1 in broiler meat: Effect of age and dietary aflatoxin B1 levels. Food Chem. Toxicol..

[8] Yarru L., Settivari R., Antoniou E., Ledoux D., Rottinghaus G. (2009). Toxicological and gene expression analysis of the impact of aflatoxin B1 on hepatic function of male broiler chicks. Poult. Sci..

[9] Fouad A.M., Ruan D., El-Senousey H.K., Chen W., Jiang S., Zheng C. (2019). Harmful effects and control strategies of aflatoxin b1 produced by Aspergillus flavus and Aspergillus parasiticus strains on poultry. Toxins.

[10] Ma R., Zhang L., Liu M., Su Y.-T., Xie W.-M., Zhang N.-Y., Dai J.-F., Wang Y., Rajput S.A., Qi D.-S. (2018). Individual and combined occurrence of mycotoxins in feed ingredients and complete feeds in China. Toxins.

[11] Li S., Muhammad I., Yu H., Sun X., Zhang X. (2019). Detection of Aflatoxin adducts as potential markers and the role of curcumin in alleviating AFB1-induced liver damage in chickens. Ecotox. Environ. Saf..

[12] Li Y., Ma Q.-G., Zhao L.-H., Wei H., Duan G.-X., Zhang J.-Y., Ji C. (2014). Effects of lipoic acid on immune function, the antioxidant defense system, and inflammation-related genes expression of broiler chickens fed aflatoxin contaminated diets. Int. J. Mol. Sci..

[13] Sun L.-H., Zhang N.-Y., Zhu M.-K., Zhao L., Zhou J.-C., Qi D.-S. (2015). Prevention of aflatoxin B1 hepatoxicity by dietary selenium is associated with inhibition of cytochrome P450 isozymes and up-regulation of 6 selenoprotein genes in chick liver. J. Nutr..

[14] Chen X., Che C., Korolchuk V.I., Gan F., Pan C., Huang K. (2017). Selenomethionine alleviates AFB1-induced damage in primary chicken hepatocytes by inhibiting CYP450 1A5 expression via upregulated SelW expression. J. Agric. Food Chem..

[15] Zhao L., Feng Y., Deng J., Zhang N.-Y., Zhang W.-P., Liu X.-L., Rajput S.A., Qi D.-S., Sun L.-H. (2019). Selenium deficiency aggravates aflatoxin B1–induced immunotoxicity in chick spleen by regulating 6 selenoprotein genes and redox/inflammation/apoptotic signaling. J. Nutr..

[16] Deng J., Zhao L., Zhang N.-Y., Karrow N.A., Krumm C.S., Qi D.-S., Sun L.-H. (2018). Aflatoxin B1 metabolism: Regulation by phase I and II metabolizing enzymes and chemoprotective agents. Mutat. Res. Rev. Mutat. Res..

[17] Murcia H.W., Diaz G.J. (2021). Protective effect of glutathione S-transferase enzyme activity against aflatoxin B1 in poultry species: Relationship between glutathione S-transferase enzyme kinetic parameters, and resistance to aflatoxin B1. Poult. Sci..

[18] Umaya S.R., Vijayalakshmi Y., Sejian V. (2021). Exploration of plant products and phytochemicals against aflatoxin toxicity in broiler chicken production: Present status. Toxicon.

[19] Muhammad I., Wang H., Sun X., Wang X., Han M., Lu Z., Cheng P., Hussain M.A., Zhang X. (2018). Dual role of dietary curcumin through attenuating AFB1-induced oxidative stress and liver injury via modulating liver phase-I and phase-II enzymes involved in AFB1 bioactivation and detoxification. Front. Pharmacol..

[20] Zhang N.-Y., Qi M., Zhao L., Zhu M.-K., Guo J., Liu J., Gu C.-Q., Rajput S.A., Krumm C.S., Qi D.-S. (2016). Curcumin prevents aflatoxin B1 hepatoxicity by inhibition of cytochrome P450 isozymes in chick liver. Toxins.

[21] Gowda N.K., Ledoux D.R., Rottinghaus G.E., Bermudez A.J., Chen Y.C. (2009). Antioxidant efficacy of curcuminoids from turmeric (Curcuma longa L.) powder in broiler chickens fed diets containing aflatoxin B1. Br. J. Nutr..

[22] Wang Y., Liu F., Liu M., Zhou X., Wang M., Cao K., Jin S., Shan A., Feng X. (2022). Curcumin mitigates aflatoxin B1-induced liver injury via regulating the NLRP3 inflammasome and Nrf2 signaling pathway. Food Chem. Toxicol..

[23] Muhammad I., Sun X., Wang H., Li W., Wang X., Cheng P., Li S., Zhang X., Hamid S. (2017). Curcumin successfully inhibited the computationally identified CYP2A6 enzyme-mediated bioactivation of aflatoxin B1 in arbor acres broiler. Front. Pharmacol..

[24] Liu X., Lin X., Zhang S., Guo C., Li J., Mi Y., Zhang C. (2018). Lycopene ameliorates oxidative stress in the aging chicken ovary via activation of Nrf2/HO-1 pathway. Aging.

[25] Xu F., Yu K., Yu H., Wang P., Song M., Xiu C., Li Y. (2017). Lycopene relieves AFB1-induced liver injury through enhancing hepatic antioxidation and detoxification potential with Nrf2 activation. J. Funct. Foods.

[26] Wan X., Ji H., Ma H., Yang Z., Li N., Chen X., Chen Y., Yang H., Wang Z. (2022). Lycopene alleviates aflatoxin B1 induced liver damage through inhibiting cytochrome 450 isozymes and improving detoxification and antioxidant systems in broiler chickens. Ital. J. Anim. Sci..

[27] Rajput S.A., Sun L., Zhang N.-Y., Khalil M.M., Ling Z., Chong L., Wang S., Rajput I.R., Bloch D.M., Khan F.A. (2019). Grape seed proanthocyanidin extract alleviates aflatoxinB1-induced immunotoxicity and oxidative stress via modulation of NF-κB and Nrf2 signaling pathways in broilers. Toxins.

[28] Lin A., Shen S., Wang J., Yan B. (2008). Reproduction diversity of Enteromorpha prolifera. J. Integr. Plant Biol..

[29] Zhong R., Wan X., Wang D., Zhao C., Liu D., Gao L., Wang M., Wu C., Nabavid S.M., Daglia M. (2020). Polysaccharides from marine Enteromorpha: Structure and function. Trends Food Sci. Technol..

[30] Guo Y., Zhao Z.-H., Pan Z.-Y., An L.-L., Balasubramanian B., Liu W.-C. (2020). New insights into the role of dietary marine-derived polysaccharides on productive performance, egg quality, antioxidant capacity, and jejunal morphology in late-phase laying hens. Poult. Sci..

[31] Liu W.-C., Zhuang D.-P., Zhao Y., Balasubramanian B., Zhao Z.-H. (2022). Seaweed-Derived Polysaccharides Attenuate Heat Stress-Induced Splenic Oxidative Stress and Inflammatory Response via Regulating Nrf2 and NF-κB Signaling Pathways. Mar. Drugs.

[32] Zhao Y., Balasubramanian B., Guo Y., Qiu S.-J., Jha R., Liu W.-C. (2021). Dietary Enteromorpha polysaccharides supplementation improves breast muscle yield and is associated with modification of mrna transcriptome in broiler chickens. Front. Vet. Sci..

[33] Liu W.-C., Ou B.-H., Liang Z.-L., Zhang R., Zhao Z.-H. (2021). Algae-derived polysaccharides supplementation ameliorates heat stress-induced impairment of bursa of Fabricius via modulating NF-κB signaling pathway in broilers. Poult. Sci..

[34] Liu W.-C., Guo Y., Zhihui Z., Jha R., Balasubramanian B. (2020). Algae-derived polysaccharides promote growth performance by improving antioxidant capacity and intestinal barrier function in broiler chickens. Front. Vet. Sci..

[35] Liu W.-C., Zhu Y.-R., Zhao Z.-H., Jiang P., Yin F.-Q. (2021). Effects of Dietary Supplementation of Algae-Derived Polysaccharides on Morphology, Tight Junctions, Antioxidant Capacity and Immune Response of Duodenum in Broilers under Heat Stress. Animals.

[36] Guo Y., Balasubramanian B., Zhao Z.-H., Liu W.-C. (2021). Marine algal polysaccharides alleviate aflatoxin B1-induced bursa of Fabricius injury by regulating redox and apoptotic signaling pathway in broilers. Poult. Sci..

[37] Livak K.J., Schmittgen T.D. (2001). Analysis of relative gene expression data using real-time quantitative PCR and the 2−ΔΔCT method. Methods.

[38] Rui X., Chen S., Li C. (2014). Protective effects of selenium on aflatoxin B1 induced hepatic oxidative stress in broilers. Chin. J. Anim. Nutr..

[39] Zhao L., Deng J., Xu Z.-J., Zhang W.-P., Khalil M.M., Karrow N.A., Sun L.-H. (2021). Mitigation of aflatoxin B1 hepatoxicity by dietary hedyotis diffusa is associated with activation of NRF2/ARE signaling in chicks. Antioxidants.

[40] Lin L., Fu P., Chen N., Gao N., Cao Q., Yue K., Xu T., Zhang C., Zhang C., Liu F. (2022). Total flavonoids of Rhizoma Drynariae protect hepatocytes against aflatoxin B1-induced oxidative stress and apoptosis in broiler chickens. Ecotox. Environ. Saf..

[41] Solis-Cruz B., Hernandez-Patlan D., Petrone V.M., Pontin K.P., Latorre J.D., Beyssac E., Hernandez-Velasco X., Merino-Guzman R., Owens C., Hargis B.M. (2019). Evaluation of cellulosic polymers and curcumin to reduce aflatoxin B1 toxic effects on performance, biochemical, and immunological parameters of broiler chickens. Toxins.

[42] Van Deursen V., Damman K., Hillege H., Van Beek A., Van Veldhuisen D., Voors A. (2010). Abnormal liver function in relation to hemodynamic profile in heart failure patients. J. Card. Fail..

[43] Ashry A., Taha N.M., Lebda M.A., Abdo W., El-Diasty E.M., Fadl S.E., Morsi Elkamshishi M. (2022). Ameliorative effect of nanocurcumin and Saccharomyces cell wall alone and in combination against aflatoxicosis in broilers. BMC Vet. Res..

[44] Zhang W., Zhang X., Zou K., Xie J., Zhao S., Liu J., Liu H., Wang J., Wang Y. (2017). Seabuckthorn berry polysaccharide protects against carbon tetrachloride-induced hepatotoxicity in mice via anti-oxidative and anti-inflammatory activities. Food Funct..

[45] Gan F., Yang Y., Chen Y., Che C., Pan C., Huang K. (2018). Bush sophora root polysaccharide could help prevent aflatoxin B1-induced hepatotoxicity in the primary chicken hepatocytes. Toxicon.

[46] Itoh K., Chiba T., Takahashi S., Ishii T., Igarashi K., Katoh Y., Oyake T., Hayashi N., Satoh K., Hatayama I. (1997). An Nrf2/small Maf heterodimer mediates the induction of phase II detoxifying enzyme genes through antioxidant response elements. Biochem. Biophys. Res. Commun..

[47] Yamamoto T., Suzuki T., Kobayashi A., Wakabayashi J., Maher J., Motohashi H., Yamamoto M. (2008). Physiological significance of reactive cysteine residues of Keap1 in determining Nrf2 activity. Mol. Cell Biol..

[48] Xu L., Zhang H., Yue H., Wu S., Yang H., Qi G., Wang Z. (2018). Low-current & high-frequency electrical stunning increased oxidative stress, lipid peroxidation, and gene transcription of the mitogen-activated protein kinase/nuclear factor-erythroid 2-related factor 2/antioxidant responsive element (MAPK/Nrf2/ARE) signaling pathway in breast muscle of broilers. Food Chem..

[49] Bao J., Ding R., Zou L., Zhang C., Wang K., Liu F., Li P., Chen M., Wan J.-B., Su H. (2016). Forsythiae fructus inhibits B16 melanoma growth involving MAPKs/Nrf2/HO-1 mediated anti-oxidation and anti-inflammation. Am. J. Chin. Med..

[50] Wang J., Yuan Z., Zhang K., Ding X., Bai S., Zeng Q., Peng H., Celi P. (2018). Epigallocatechin-3-gallate protected vanadium-induced eggshell depigmentation via P38MAPK-Nrf2/HO-1 signaling pathway in laying hens. Poult. Sci..

