# Supplementary material for: Evaluation of Hepatic Detoxification Effects of Enteromorpha prolifera Polysaccharides against Aflatoxin B1 in Broiler Chickens
Source: Antioxidants (Basel). 2022 Sep 6;11(9):1757. doi: 10.3390/antiox11091757 (PMC9495745; doi:10.3390/antiox11091757)
Supplement: Supplementary file 1 [file antioxidants-11-01757-s001.zip › Table S1.pdf]

Table S1. The chemical and monosaccharide composition of seaweed-derived polysaccharides (SDP) from *Enteromorpha prolifera*.

**Items**	**Contents, %**
**Chemical composition**	
Total polysaccharides	53.32
Protein	1.65
Sulfate	19.87
Uronic acid	12.66
Moisture	3.58
Unspecified compounds (lipid, salt, etc.)	8.92
**Monosaccharide composition**	
Glucosamine (GlcN)	50.81
Glucose (Glc)	27.70
Galacturonic acid (GalA)	11.75
Mannose (Man)	2.78
Xylose (Xyl)	2.63
Galactose (Gal)	1.95
Arabinose (Ara)	0.93
Glucuronic acid (GlcA)	0.76
Fucose (Fuc)	0.27
Galactosamine (GalN)	0.25
Ribose (Rib)	0.17

A phenol-sulfuric acid assay was used to determine the total polysaccharide content and Glc was used as a standard. Bradford's method was used to analysis the protein content and bovine serum albumin (BSA) was used as a standard. The barium sulfate-gelatin turbidimetric method was used to determine the sulfate content and potassium sulfate was used as a standard. m-Hydroxybiphenyl colorimetry was used to measure uronic acid content and d-GlcA was used as a standard. Monosaccharide composition was determined using high-performance liquid chromatography (HPLC).
