# Supplementary material for: Evaluation of Hepatic Detoxification Effects of Enteromorpha prolifera Polysaccharides against Aflatoxin B1 in Broiler Chickens
Source: Antioxidants (Basel). 2022 Sep 6;11(9):1757. doi: 10.3390/antiox11091757 (PMC9495745; doi:10.3390/antiox11091757)
Supplement: Supplementary file 1 [file antioxidants-11-01757-s001.zip › Table S2.pdf]

Table S2. The formulation and nutrient levels of the basal diet.

Item	Contents (%)
**Ingredients**	
Corn	60.84
Soybean meal	32.11
Wheat bran	2.16
Soybean oil	2.00
Limestone	1.28
$CaHPO_4$	1.26
DL-Methionine	0.15
Vitamin premix[1]	0.10
Mineral premix[2]	0.10
Total	100.00
**Nutrient levels[3]**	
ME (MJ/kg)	11.94
Crude protein (%)	18.22
Ca (%)	0.98
Met (%)	0.32
Cystine (%)	0.31
Lys (%)	0.90
Total phosphorus (%)	0.51

[1]Premix provided per kilogram of diet: 5,000 IU of vitamin A, 1000 IU of vitamin D_3, 10 IU of vitamin E, 0.5 mg of vitamin K_3, 3 mg of thiamin, 7.5 mg of riboflavin, 4.5 mg of vitamin B_6, 10 μg of vitamin B_{12}, 25 mg of niacin, 0.55 mg of folic acid, 0.2 mg of biotin, 500 mg of choline, and 10.5 mg of pantothenic acid.

[2]Premix provided per kilogram of diet: 60 mg of Zn, 80 mg of Mn, 80 mg of Fe, 3.75 mg of Cu, 0.35 mg of I, and 0.15 mg of Se.

[3]Except for metabolic energy (ME), others are measured values.
